# Exosome-mediated lncRNA SND1-IT1 from gastric cancer cells enhances malignant transformation of gastric mucosa cells via up-regulating SNAIL1

**DOI:** 10.1186/s12967-022-03306-w

**Published:** 2022-06-23

**Authors:** Guohua Jin, Jianguang Zhang, Tingting Cao, Bang Chen, Yu Tian, Yang Shi

**Affiliations:** 1 Department of Gastric & Intestine, The First Hospital of Jilin University, No.1 Xinmin Street, Changchun, 130021 Jilin China; 2 Department of Gastroenterology, The Sixth Affiliated Hospital of Xinjiang Medical University, Urumqi, 830002 Xinjiang China

**Keywords:** Gastric cancer, SND1-IT1, Exosome, USP3, Deubiquitination

## Abstract

**Background:**

Gastric cancer (GC), as one of the most common malignancies across the globe, is the fourth leading cause of cancer-related deaths. Though a large body of research has been conducted to develop the therapeutic methods of GC, the survival rate of advanced patients is still poor. We aimed to dig into the potential regulatory mechanism of GC progression.

**Methods:**

Bioinformatics tools and fundamental assays were performed at first to confirm the candidate genes in our study. The functional assays and mechanism experiments were conducted to verify the regulatory mechanisms of the genes underlying GC progression.

**Results:**

Long non-coding RNA (lncRNA) SND1 intronic transcript 1 (SND1-IT1) is highly expressed in exosomes secreted by GC cells. SND1-IT1 was verified to bind to microRNA-1245b-5p (miR-1245b-5p) through competitive adsorption to promote ubiquitin specific protease 3 (USP3) messenger RNA (mRNA) expression. SND1-IT1 was validated to recruit DEAD-box helicase 54 (DDX54) to promote USP3 mRNA stability. SND1-IT1 induces malignant transformation of GES-1 cells through USP3. USP3 mediates the deubiquitination of snail family transcriptional repressor 1 (SNAIL1).

**Conclusions:**

Exosome-mediated lncRNA SND1-IT1 from GC cells enhances malignant transformation of GES-1 cells via up-regulating SNAIL1.

**Graphical Abstract:**

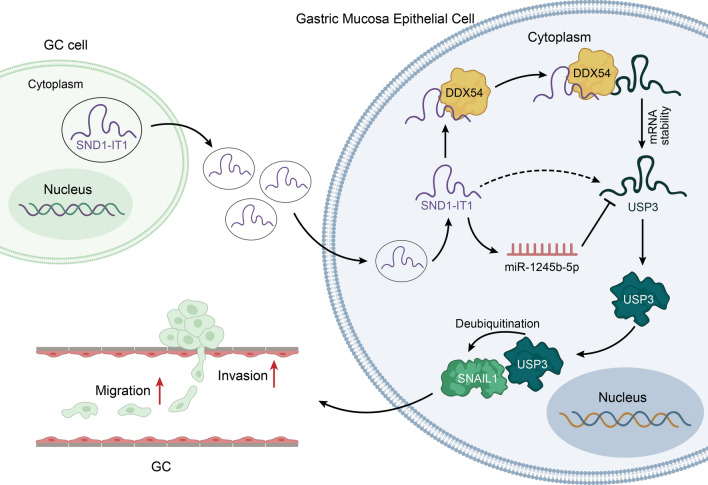

**Supplementary Information:**

The online version contains supplementary material available at 10.1186/s12967-022-03306-w.

## Background

Gastric cancer (GC) is one of the most common digestive malignant tumors [1]. As much progress has been made in surgical techniques, the 5-year survival rate of early GC can reach > 95%. However, it’s difficult to make timely diagnosis, which means that most patients are diagnosed at advanced stage, missing the opportunity for surgery. Therefore, we aimed to deepen the understanding of the mechanism underlying GC for improving its treatment.

Long non‑coding RNAs (lncRNAs) are defined as a type of non-coding RNAs with over 200 nucleotides in length. Most of lncRNAs are generated from gene introns, intergenic regions, promoter regions of coding messenger RNA (mRNA), antisense strands of mRNAs and pseudogenes [2]. More importantly, lncRNAs have been widely reported to function as potential biomarkers in modulating a variety of biological and pathological processes, including GC progression. For instance, lncRNA MEG3 inhibits proliferation and metastasis of GC through targeting p53 signaling pathway [3]; ALKBH5 propels invasion and metastasis of GC via hampering methylation of the lncRNA NEAT1 [4]; lncRNA AK023391 facilitates tumorigenesis and invasion of GC by activating PI3K/Akt signaling pathway [5]. By using Gene Expression Omnibus (GEO) database, we found that lncRNA SND1-IT1 was highly expressed in the exosomes from plasma of GC patients. However, its effects on GC progression have rarely been reported. Therefore, the present study focuses on the role of SND1-IT1 in GC.

MicroRNAs (miRNAs) are small non-coding RNAs, which down-regulate gene expression by repressing or degrading target mRNAs [6]. Moreover, mRNA-based therapeutics has been widely applied in preclinical and clinical studies for cancer treatment [7]. It has been reported that the competing endogenous RNA (ceRNA) network (lncRNA-miRNA-mRNA) has been identified in various cancers including squamous cell carcinoma [8], melanoma [9] and lung adenocarcinoma [10]. In addition, the ceRNA network has also been widely studied in GC. For example, LINC01133 as a ceRNA inhibits the progression of GC via sequestering miR-106a-3p to modulate APC expression and the Wnt/β-catenin pathway [11]; MT1JP as a ceRNA regulates FBXW7 via competitively binding to miR-92a-3p in GC [12]; HOTAIR functions as a ceRNA to modulate the expression of HER2 via sequestering miR-331-3p in GC [13].

According to the previous study, it has been identified that lncRNA plays an important role through the interaction with RNA-binding proteins (RBPs) in various cancer cells [14]. The functions and roles of RBPs in GC have already been studied. However, the RBPs which can bind to SND1-IT1 in GC cells still remain to be disclosed.

To summarize, the main focus point of our research was to study the underlying mechanisms by which SND1-IT1 affects the progression of GC.

## Methods

### Cell culture

Human gastric mucosa epithelial cell line GES-1 was commercially acquired from Shanghai EK-Bioscience Biotechnology Co., LTD (Shanghai, China). Cells were cultured in Dulbecco's Modified Eagle's Medium (DMEM) + 10% fetal bovine serum (FBS). Human GC cell line AGS was procured from America Type Culture Collection (ATCC) and cultured in F-12 K medium + 10% FBS. HEK-293 T cells were procured from ATCC and cultured in DMEM + 10% FBS + 2 mM glutamine. All cells were preserved at 37 °C with 5% CO_2_.

### Plasmid transfection

For the overexpression of SND1-IT1 and USP3, the full-length sequences were separately synthesized and sub-cloned into pcDNA3.1 vectors. Empty pcDNA3.1 vector was utilized as the negative control (NC). MiR-1245b-5p was overexpressed using miR-1245b-5p mimics. For the inhibition of DDX54 and SND1-IT1, specific small interfering RNAs (siRNAs) were respectively synthesized and established with non-targeting siRNA (si-NC) as NC.

### Bioinformatics analysis

GEO database is a public functional genomics data repository supporting MIAME-compliant data submissions. GEO database (GSE153413) was used in our study to explore lncRNAs significantly overexpressed in exosomes from the plasma of GC patients. UALCAN database (http://ualcan.path.uab.edu/index.html) is a web resource for the analysis of cancer OMICS data, which was utilized in our study to predict the expressions of ZNF529-AS1, LIMD1-AS1, LINC00837, SND1-IT1, VWA8-AS1, LINC01005, ST20-AS1, LINC00511 and LINC01266 in stomach adenocarcinoma (STAD). StarBase (https://rna.sysu.edu.cn/encori/index.php) is designed to decode the interaction networks; it was used in our study to predict the miRNAs binding to SND1-IT1, mRNAs binding to miR-1245b-5p and their expressions, RBPs binding to both SND1-IT1 and USP3 and interaction between DDX54 and USP3 3′UTR. BioGRID (https://thebiogrid.org/) is an online interaction repository with data compiled through comprehensive curation efforts, which was used to predict the proteins interacting with USP3.

### Quantitative polymerase chain reaction (q-PCR)

The RNA expressions of SND1-IT1, VWA8-AS1, LINC00511, matrix metallopeptidase 2 (MMP2), matrix metallopeptidase 9 (MMP9), miR-873-3p, miR-1245b-5p, USP3, IGF2BP1, TKT, DDX54, β-actin and SNAIL1 were detected by q-PCR. Total RNA was extracted from GC cell lines using Trizol. Afterwards, the extracted RNAs were converted into 1μL complementary DNA (cDNA) through reverse transcription reaction with the adoption of PrimeScript™ RT reagent kit. Subsequently, q-PCR was conducted using SYBR Green PCR Master Mix. The results were calculated based on 2^−∆∆Ct^. GAPDH was utilized as internal reference. The primers used in the assays were supplemented.

### Western blot

Western blot was conducted to estimate the protein levels of CD63, CD81, CD9, DDX54, ISLR, SNAIL1, CHEK1, β-actin and USP3. Total protein extracted from GC cell lines was subjected to isolation using RIPA buffer, followed by being separated via sodium dodecyl sulfate–polyacrylamide gel electrophoresis (SDS-PAGE). Subsequently, the samples were transferred to polyvinylidene fluoride (PVDF) membranes and blocked in 5% skim milk. The membranes were then cultivated with primary antibodies overnight at 4 °C. After being washed in TBST, the samples were incubated with the secondary antibodies. Finally, the results were visualized by enhanced chemiluminescence (ECL) substrate.

### Transwell assay

Transwell assays were conducted for the evaluation of GC cell migration and invasion. Transfected cells were planted into the upper chambers of 24-well transwell chambers at a density of 2 × 10^4^ cells per well. The chambers coated with Matrigel were applied for the implementation of invasion assay while ones without Matrigel for migration assay. Complete medium was added into the lower chambers. Twenty-four hours later, cells in the upper chambers were abraded with caution using a cotton swab and cells in the lower chambers were fixed in methanol solution for 15 min. Crystal violet was adopted to stain the membranes for 10 min. The invaded or migrated cells were observed and calculated under a microscope (10 × 10).

### RNA-binding protein immunoprecipitation (RIP) assay

RIP assay was performed using Magna RIP™ RNA-Binding Protein Immunoprecipitation Kit. To set up immunoglobulin G (IgG), Argonaute 2 (AGO2) and DDX54 groups, 50 μg Protein A/G Agarose magnetic beads were incubated with the antibodies against AGO2, IgG and DDX54 overnight at 4 °C through rotation. 6 × 10^7^ gastric cells were collected, followed by lysis in Immunoprecipitation (IP) Lysis Buffer (Beyotime Biotechnology Co., LTD). 100μL lysis buffer was added to IgG and DDX54 groups respectively, and 10 μL to the Input group. Next, lysates were cultured with anti-DDX54 antibody and anti-IgG antibody (Abcam) overnight at 4 °C in rotation. Finally, the Imprint® RNA Immunoprecipitation Kit (RIP-12RXN, Sigma-Aldrich, USA) was employed to purify and extract the RNA precipitates.

### RNA pull-down assay

In brief, a single biotinylated desthiobiotinylated cytidine was attached to 3′end of RNAs (SND1-IT1, miR-1245b-5p and USP 3′UTR). After reaching a final concentration of 20 nmol/L, the biotinylated RNAs were subjected to co-cultivation with streptavidin-coated magnetic beads (Ambion, Life Technologies). Afterwards, the beads were incubated with the cell lysate. Western blot and qRT-PCR was performed to analyze the abundance of DDX54 in bound fractions after the pull-down of biotin-coupled RNA complex.

### Dual-luciferase reporter assay

For luciferase reporter assay, full-length sequence of SND1-IT1 or USP3 3′UTR possessing wild-type and mutant miR-1245b-5p binding sites were sub-cloned into pmirGLO luciferase vectors for the construction of pmirGLO-SND1-IT1-WT/MUT and pmirGLO-USP3 3′UTR-WT/MUT. MiR-1245b-5p mimics or mimics NC were co-transfected with pmirGLO-SND1-IT1-WT/MUT or pmirGLO-USP3 3′UTR-WT/MUT into 293 T cells. After 48-h transfection, the luciferase activities were analyzed utilizing the dual-luciferase reporter assay system (Promega).

### Wound healing assay

In the wound healing assay, the 5 × 10^5^ transfected GES-1 cells were seeded in 24-well plates and cultivated at 37 °C until cells reached 100% confluence. Next, cells were scraped by 200 μL sterile micropipette tip, followed by being cultured at 37 °C for 24 h. Afterwards, the cells were washed three times in serum-free medium for the removal of the detached cells. The scratch was imaged via microscopy at the time 0 h and 24 h for analysis.

### Co-immunoprecipitation (Co-IP) assay

The prepared cell lysates were collected from the treated cells in IP lysis buffer, followed by incubation with indicated antibodies of SNAIL1 and USP3 and control IgG antibody overnight at 4 °C. After being mixed with beads, samples were washed in IP lysis buffer, followed by analysis of western blot.

### In vivo xenograft experiments

Nude mice aged 6–8 weeks were randomly divided into 2 groups (n = 5). Each group received subcutaneous injection of exosomes Exo/pcDNA3.1 or Exo/SND1-IT1 (1 × 10^9^ exosomes/ml). Seven days after the injection, the tumor volume was measured every 3 days. Twenty-eight days after the injection, all the mice were sacrificed for tumor resection, followed by the measurement of tumor weight. The animal studies were implemented with the approval of the First Hospital of Jilin University.

### Statistical analysis

Statistical analyses for separate and triplicated experiments were conduct using SPSS version 17.0. The experimental data were presented as mean ± standard deviation (SD). The differences between two groups were subjected to analysis by the Student’s *t*-test, while the differences between more than two groups by one-way or two-way analysis of variance (ANOVA). P value under 0.05 was considered to be the criterion for statistical significance.

## Results

### GC cells induce the malignant progression of GES-1 cells via exosomes

**Fig. 1:**
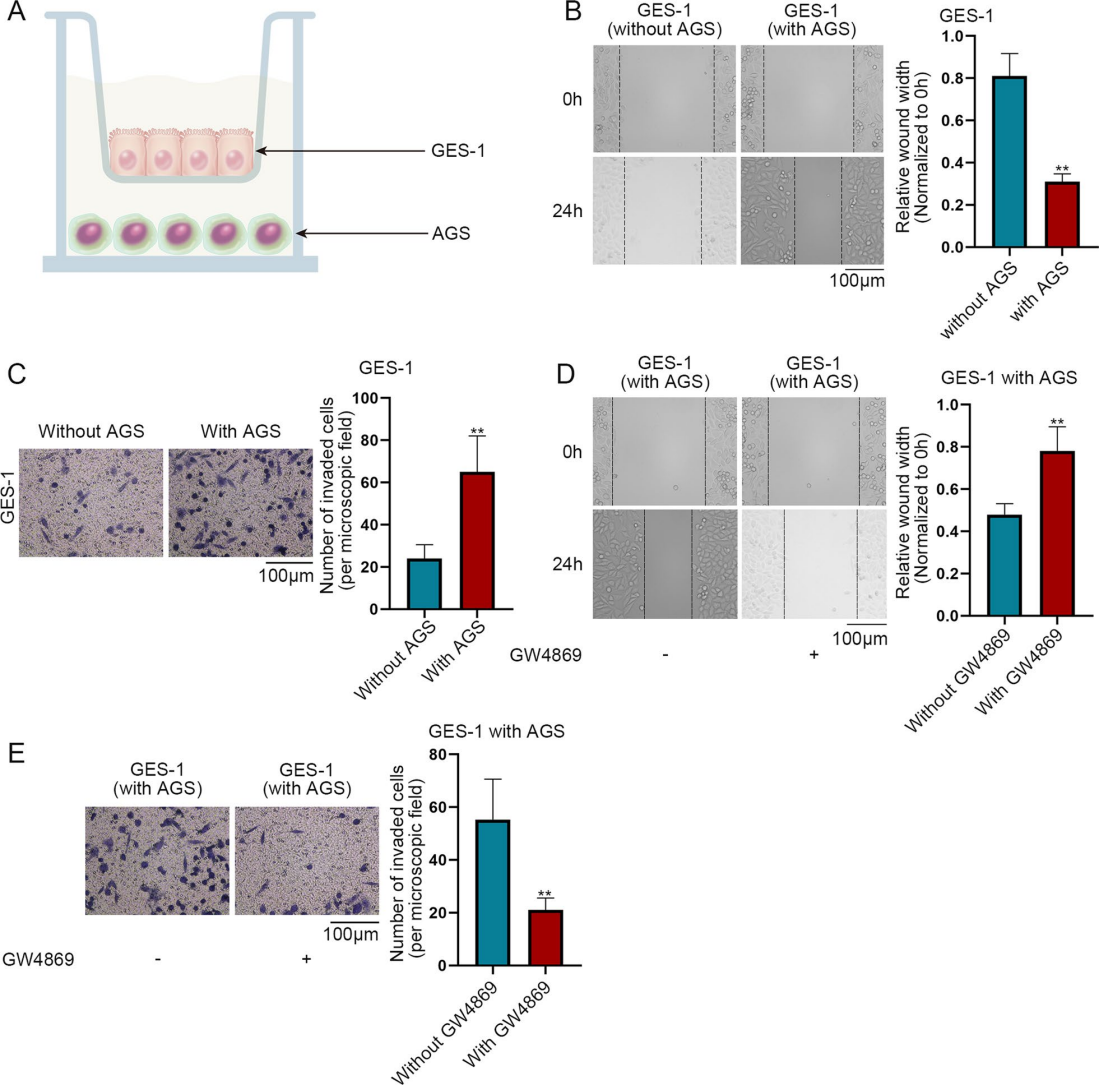
GC cells induce malignant progression of GES-1 cells via exosomes. **A** GES-1 cells were co-cultured with AGS cells. **B** GES-1 cell migratory before and after being co-cultured with AGS cells was detected by wound healing assay. **C** Transwell assay was performed to detect the invasive capability of GES-1 cell before and after being co-cultured with AGS cells. **D** Wound healing assay was performed to measure the migration of AGS-cultured GES-1 cells before and after treatment with GW4869. **E** Transwell assay was taken to determine AGS-cultured GES-1 cell invasion before and after treatment with GW4869. ^**^P < 0.01

It was reported that in the tumor microenvironment (TME), cancer cells induce the carcinogenesis of normal cells [15]. Hence, we performed assays to explore whether GC cells have the same influence on gastric mucosa epithelial cells. At first, we co-cultured GC cell line AGS with gastric mucosa epithelial cell line GES-1 (Fig. 1A). Secondly, the GES-1 cell migratory capacity before and after the co-culture was detected by wound healing assay. The result showed that in GES-1 cells with AGS, the wound width was narrower than that in GES-1 cells without AGS, which indicated that the co-culture with AGS cells promotes GES-1 cell migration (Fig. 1B). Thirdly, transwell assay was performed to detect the cell invasive capability. After co-culture with AGS, GES-1 cell invasion was promoted (Fig. 1C). It has been reported that cancer cells can regulate the malignant progression of normal epithelial cells through secreting exosomes [16]. Hence we speculated that GC cells can influence GES-1 cells via secreting exosomes. GW4869, with its ability to inhibit exosome secretion, was adopted [17]. AGS cells, with and without the treatment of GW4869 were then co-cultured with GES-1 cells respectively. The results of wound healing assay showed that cell migration in co-cultured cells was suppressed after GW4869 treatment (Fig. 1D). It was unearthed by the results of transwell assay that invasive ability of GES-1 cells was attenuated after GW4869 treatment in the co-culture system (Fig. 1E). To conclude, it was verified that GC cells promotes GES-1 cell migration and invasion via secreting exosomes.

### GC cells secrete SND1-IT1 via exosomes

**Fig. 2:**
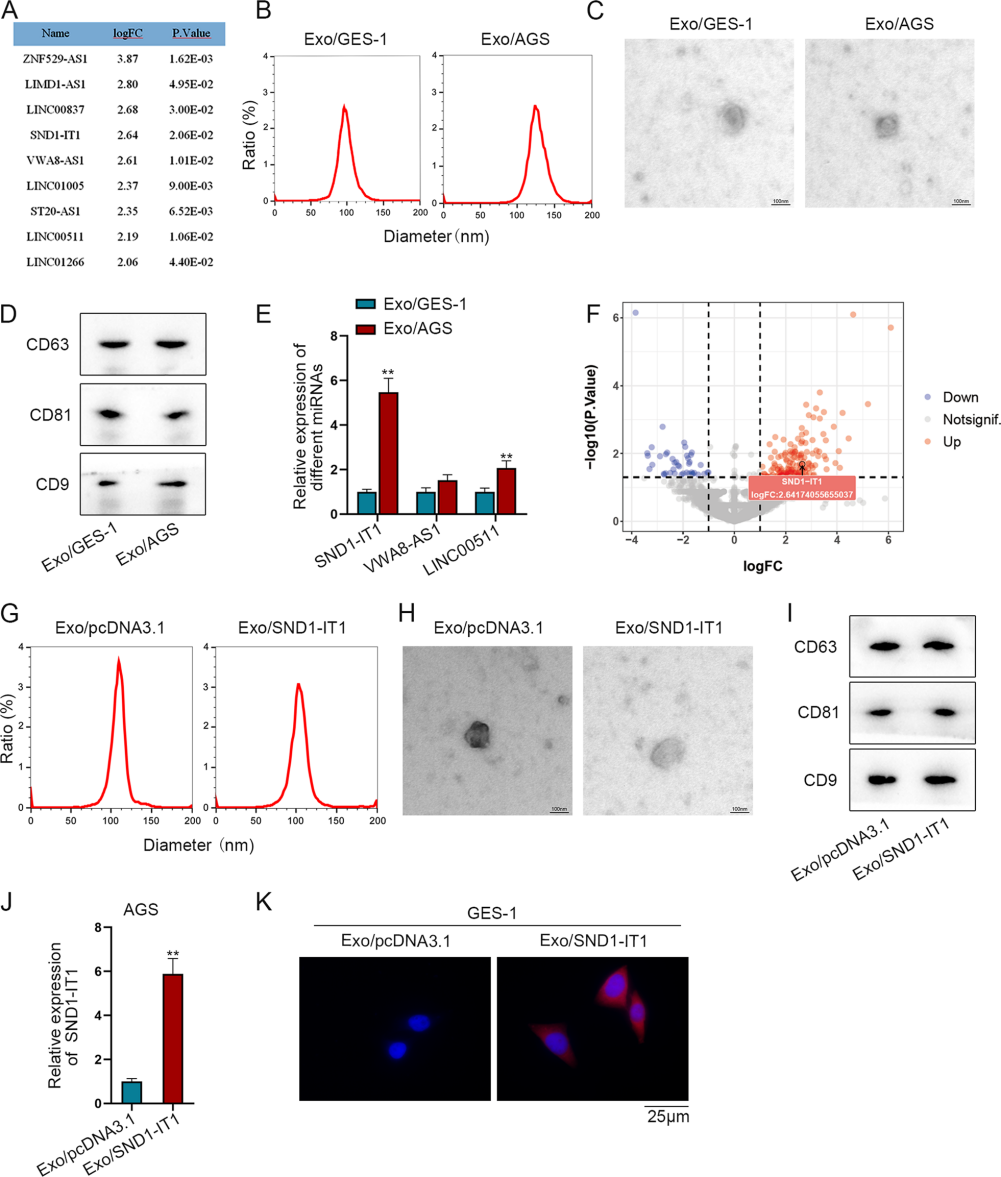
GC cells secrete SND1-IT1 via exosomes. **A** The list of lncRNAs significantly overexpressed in plasma exosomes of GC patients was screened by GSE153413 dataset under the condition of p < 0.05 and logFC > 2. **B** NTA detection was taken to examine the diameters of Exo/AGS and Exo/GES-1. **C** The morphology of exosomes was observed by an electron microscope. **D** Western blot was performed to detect the expression of CD63, CD81 and CD9. E. SND1-IT1, VWA8-AS1 and LINC00511 expressions were measured by q-PCR in Exo/AGS and Exo/GES-1. F. The expression of SND-IT1 in the plasma exosomes of GC patients was analyzed through GSE153414. **G** NTA detection was taken to examine the diameters of Exo/pcDNA3.1 and Exo/SND1-IT1. **H** The morphology of Exo/pcDNA3.1 and Exo/SND1-IT1 was observed by electron microscope. **I** Western blot was conducted to detect the protein levels of CD63, CD81 and CD9 in Exo/pcDNA3.1 and Exo/SND1-IT1. **J** The expression of SND1-IT1 in Exo/pcDNA3.1 and Exo/SND1-IT1 was detected by q-PCR. **K** GES-1 cells were treated with Exo/pcDNA3.1 and fluorescent-labeled Exo/SND1-IT1 respectively, and the cell fluorescence was observed under a fluorescence microscope. ^**^P < 0.01

From Fig. 1 we realized that GC cells can induce malignant transformation of gastric mucosa epithelial cells. Next, we dug into the specific mechanisms underlying induction. By utilizing GEO database (GSE153413), lncRNAs significantly overexpressed in exosomes from the plasma of GC patients were screened out under the conditions of p < 0.05 and logFC > 2. Nine overexpressed lncRNAs were listed as shown in Fig. 2A. Then UALCAN database was used to analyze the expressions of the lncRNAs in STAD (Additional file 1: Fig. S1). SND1-IT1, VWA8-AS1 and LINC00511 were chosen as candidates. SND1-IT1 was reported to promote the proliferation and migration of osteosarcoma [18], while VWA8-AS1 and LINC00511 have never been reported previously.

The exosomes were extracted from AGS and GES-1 cells, and named as Exo/AGS and Exo/GES-1 respectively. Nanoparticle tracking analysis (NTA) was implemented to examine the diameter of Exo/AGS and Exo/GES-1. The result showed that the diameter of the extracted exosomes was about 60–160 nm (Fig. 2B). Afterwards, morphology of exosomes was observed by electron microscope, which is in accordance with the basic characteristics of exosomes (Fig. 2C). Western blot assay was then performed to detect the protein level of CD63, CD81 and CD9, showing their existence (Fig. 2D). The results from Fig. 2B–D all demonstrated that exosomes were successfully extracted. Subsequently, the expressions of candidate lncRNAs were measured by q-PCR in Exo/AGS and Exo/GES-1. As SND1-IT1 expression was most up-regulated among other candidates, it was chosen for the following experiments (Fig. 2E). The expression of SND-IT1 in the exosomes from the plasma of GC patients was analyzed through GSE153414 dataset. As shown in Fig. 2F, SND-IT1 was predicted to be up-regulated in the exosomes from the plasma. Next, SND1-IT1 was overexpressed by transfection with pcDNA3.1-SND1-IT1 vector, the efficiency of which was detected by q-PCR in AGS and GES-1 cells (Additional file 2: Fig. S2A, B). Exosomes extracted from GC cells transfected with pcDNA3.1 and pcDNA3.1-SND1-IT1 were termed as Exo/pcDNA3.1 and Exo/SND1-IT1 respectively. Similarly, the diameter and morphology of these exosomes were examined (Fig. 2G, H). The protein levels of CD63, CD81 and CD9 were detected by western blot in these exosomes (Fig. 2I). The results from Fig. 2G–I demonstrated that GC cell-derived exosomes were extracted successfully. The expression of SND1-IT1 in AGS cells treated with Exo/pcDNA3.1 or Exo/SND1-IT1 was detected by q-PCR. The results showed that the expression of SND1-IT1 in exosomes was up-regulated in Exo/SND1-IT1 group, compared with control group (Fig. 2J). Next, GES-1 cells were treated with Exo/pcDNA3.1 and fluorescent-labeled Exo/SND1-IT1 respectively, followed by observation under fluorescence microscope. It was proved that SND1-IT1 can be delivered through GC cell-derived exosomes into GES-1 cells (Fig. 2K). Taken together, GC cells secrete exosomal SND1-IT1 into GES-1 cells.

### Exosomal SND1-IT1 promotes GES-1 cell migration and invasion

**Fig. 3:**
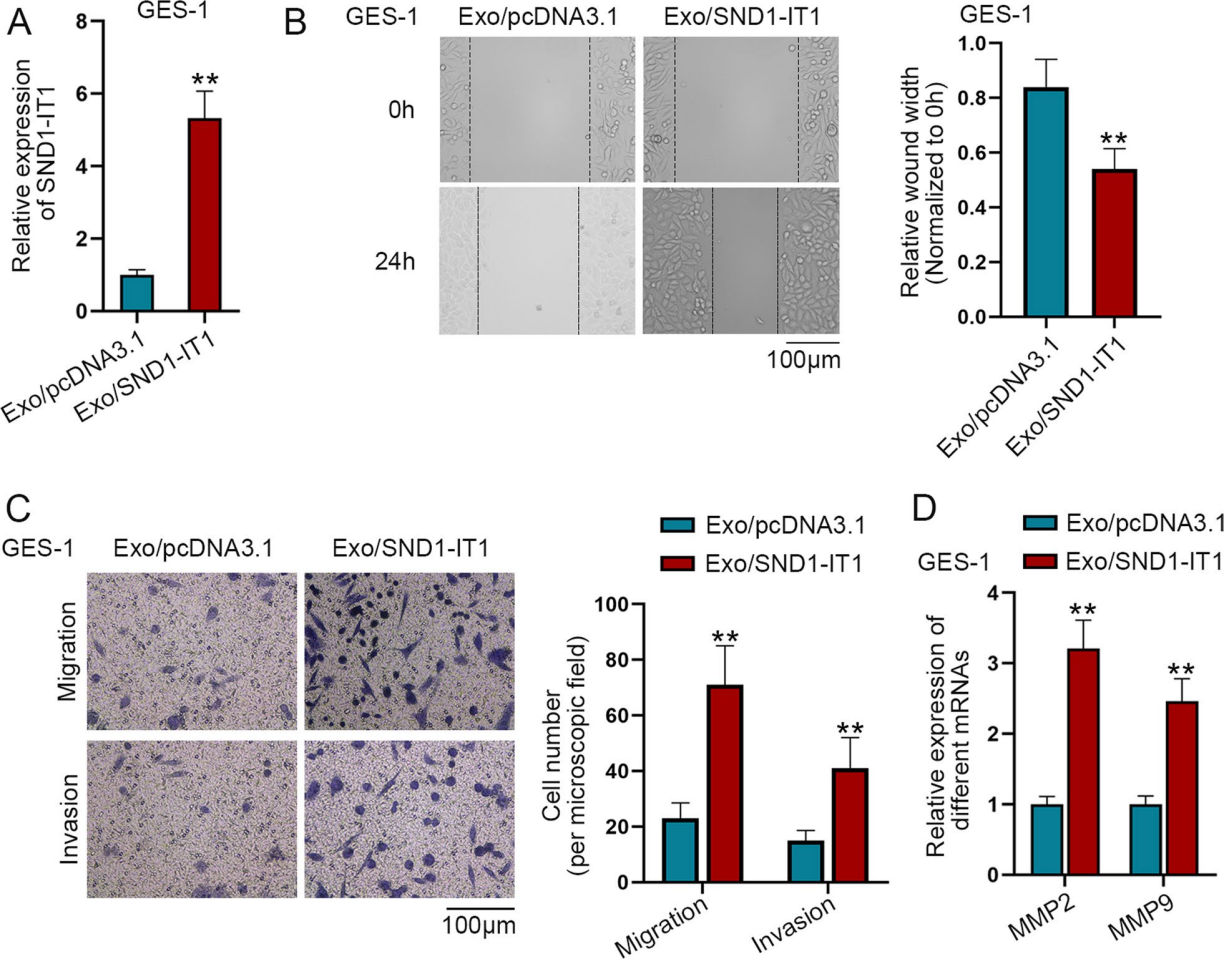
Exosomal SND1-IT1 promotes migration and invasion of GES-1 cells. GES-1 cells were treated with Exo/pcDNA3.1 and Exo/SND1-IT1. **A** The expression of SND1-IT1 was detected by q-PCR in GES-1 cells. **B** Cell migration was measured by wound healing assay in GES-1 cells. **C** GES-1 cell migration and invasion was examined by transwell assay. **D** The expression of MMP2 and MMP9 was detected by q-PCR in GES-1 cells. ^**^P < 0.01

In this section, we decided to explore the effect of exosome-mediated SND1-IT1 on GES-1 cells. The GES-1 cells were treated with Exo/pcDNA3.1 or Exo/SND1-IT1 respectively. Next, q-PCR was utilized to detect SND1-IT1 expression, showing an increase in Exo/SND1-IT1 group compared to Exo/pcDNA3.1 group (Fig. 3A). Wound healing assay and transwell assays were conducted to measure the migratory and invasive capabilities of GES-1 cells. It was found that exosomal SND1-IT1 promoted GES-1 cell migration and invasion (Fig. 3B, C). At last, the expression levels of MMP2 and MMP9 were detected by q-PCR in GES-1 cells. MMP2 and MMP9 are invasion-related factors, and their expressions are positively related to cell invasion. The results of q-PCR showed that, after treatment with Exo/SND1-IT1, the levels of MMP2 and MMP9 were increased, suggesting that exosomal SND1-IT1 promotes GES-1 cell invasion (Fig. 3D). Taken together, exosomal SND1-IT1 promotes GES-1 cell migration and invasion.

### SND1-IT1 competitively adsorbs miR-1245b-5p to elevate USP3 expression

**Fig. 4:**
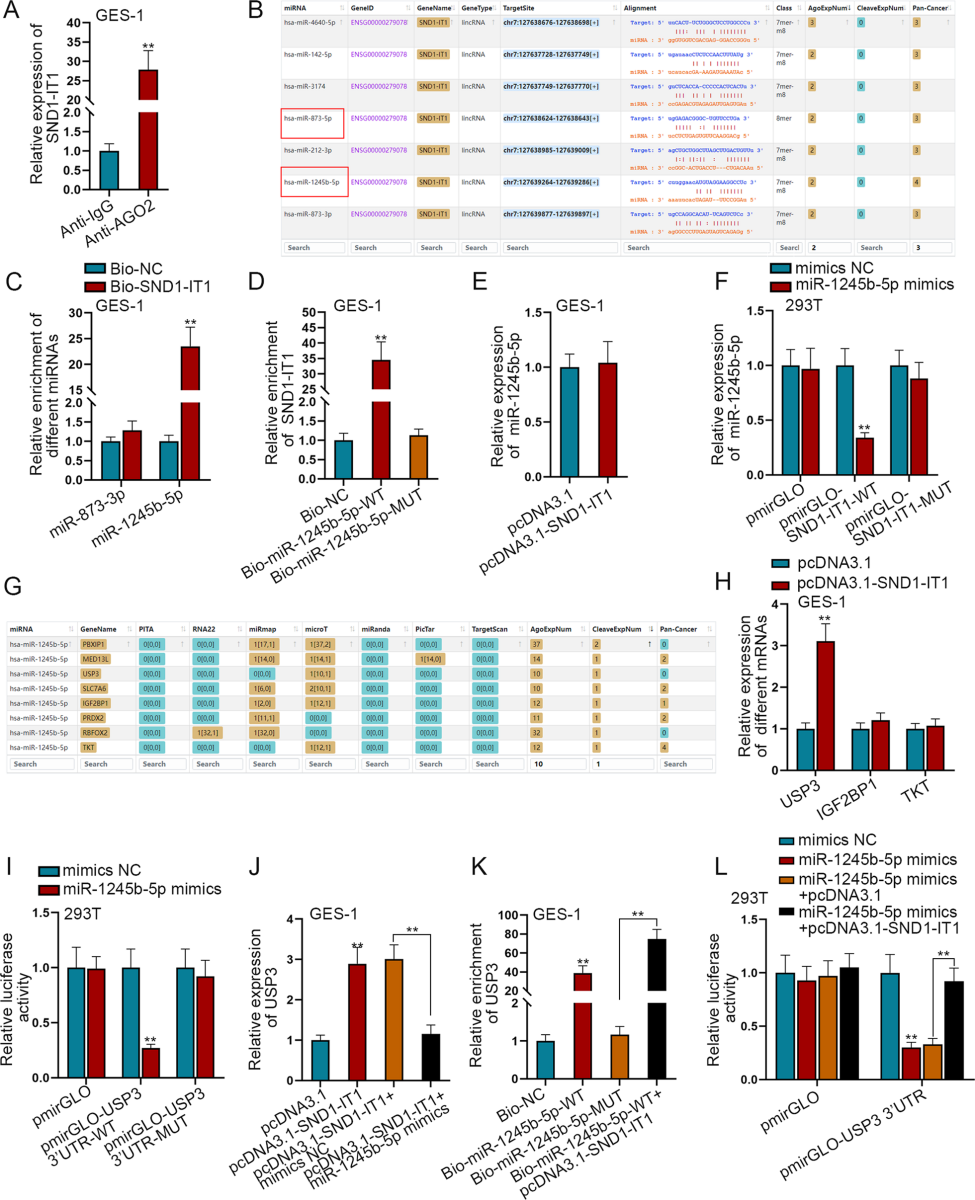
SND1-IT1 competitively adsorbs miR-1245b-5p to up-regulate USP3 expression. **A** SND1-IT1 enrichment in Anti-AGO2 group was detected by RIP assay in GES-1 cells. **B** The miRNAs which can bind to SND1-IT1 were predicted by starBase (https://rna.sysu.edu.cn/encori/index.php) when AgoExpNum ≥ 2 and Pan-Cancer ≥ 3. **C** In GES-1 cells, the interaction of SND1-IT1 with miR-873-3p and miR-1245b-5p was detected by RNA pull-down assay. **D** The interaction between SND1-IT1 and miR-1245b-5p was detected by RNA pull-down assay. **E** The expression of miR-1245b-5p was detected by q-PCR after SND1-IT1 was overexpressed. **F** In 293 T cells, dual-luciferase reporter assay detected the interaction between SND1-IT1 and miR-1245b-5p. **G** The mRNA which can bind to miR-1245b-5p was predicted and screened by starBase database. **H** In GES-1 cells, q-PCR detected the expression of candidate mRNAs before and after up-regulation of SND1-IT1. **I** In 293 T cells, dual-luciferase reporter assay detected the interaction between USP 3′UTR and miR-1245b-5p. **J** The expression level of USP3 was detected by q-PCR in GES-1 cells after the transfection of pcDNA3.1, pcDNA3.1-SND1-IT1, pcDNA3.1-SND1-IT1 + mimics NC or pcDNA3.1-SND1-IT1 + miR-1245b-5p mimics. **K** The interaction between USP3 and miR-1245b-5p was detected in GES-1 cells by RNA pull-down assay before and after SND1-IT1 overexpression. **L** In 293 T cells, dual-luciferase reporter assay was performed to detect the interaction between USP3 3′UTR and miR-1245b-5p before and after SND1-IT1 overexpression. ^**^P < 0.01

Next, we investigated the specific regulatory mechanism of SND1-IT1 in GES-1 cells through the following experiments. It was found that SND1-IT1 can regulate the progression of osteoblast via ceRNA pattern [18]. We conjectured that in GES-1 cells, exosomal SND1-IT1 regulates downstream genes by competitively adsorbing miRNA. In GES-1 cells, SND1-IT1 expression was detected by q-PCR after RIP assay. It was found that SND1-IT1 existed in RNA-induced silencing complex (RISC) as evidenced by the preferential enrichment of SND1-IT1 in Anti-AGO2 group, which indicated that SND1-IT1 might regulate downstream gene expression via miRNA (Fig. 4A). The miRNAs which can bind to SND1-IT1 were predicted by starBase under the certain conditions (AgoExpNum ≥ 2 and Pan-Cancer ≥ 3). Seven miRNAs were screened out and listed as shown in Fig. 4B. Among them, miR-1245b-5p and miR-873-3p were chosen as candidates as they had not been studied in GC. According to the literature review, miR-873-3p can inhibit lung cancer progression [19], and there are few researches on the role of miR-1245b-5p in cancers. RNA pull-down assay in GES-1 cells was performed to detect the interaction of SND1-IT1 with miR-873-3p and miR-1245b-5p. As evidenced by the higher enrichment, miR-1245b-5p had a better binding ability to SND1-IT1 than miR-873-3p (Fig. 4C). Thus miR-1245b-5p was selected for further study. For verification, RNA pull-down assay proved that SND1-IT1 can bind to miR-1245b-5p (Fig. 4D). After SND1-IT1 was overexpressed, q-PCR was performed to detect miR-1245b-5p expression. It was shown that the expression of miR-1245b-5p had no obvious change after SND1-IT1 overexpression, indicating that SND1-IT1 competitively adsorbs miR-1245b-5p (Fig. 4E). In 293 T cells, dual-luciferase reporter assay was conducted to detect the luciferase activity under different conditions. After co-transfection with miR-1245b-5p mimics, the luciferase activity in pmirGLO-SND1-IT1-WT group was decreased, further proving the interaction between miR-1245b-5p and SND1-IT1 (Fig. 4F). The mRNA which can bind to miR-1245b-5p was predicted and screened by starBase database under the condition of AgoExpNum ≥ 10 and CleaveExpNum ≥ 1 (Fig. 4G). Furthermore, the expressions of predicted mRNAs in STAD were analyzed by starBase (Additional file 2: Fig. S2C–J). USP3, SLC7A6, IGF2BP1 and TKT were chosen as candidates because they were predicted to be highly expressed in STAD. According to the literature review, USP3 promotes GC cell migration and invasion [20]; SLC7A6 hasn’t been reported in studies related to cancer; IGF2BP1 accelerates GC progression [21]; and TKT facilitates breast cancer metastasis [22]. In GES-1 cells, q-PCR was performed to detect the expression of candidate mRNAs after up-regulation of SND1-IT1. Among the candidates, USP3 expression was most increased after SND1-IT1 overexpression (Fig. 4H), thus it was selected for our study. The overexpression efficiency of miR-1245b-5p mimics was assessed by q-PCR as shown in Additional file 3: Fig. S3A. Afterwards, dual-luciferase reporter assay in 293 T cells was performed to detect the interaction between USP3 3′UTR and miR-1245b-5p. It was shown that when miR-1245b-5p was overexpressed, the luciferase activity of pmirGLO-USP3 3′UTR-WT group was decreased, while that of mutant groups had no significant change compared with control group (Fig. 4I). Subsequently, rescue experiment was conducted. We performed q-PCR to detect the expression of USP3 after the transfection of pcDNA3.1, pcDNA3.1-SND1-IT1, pcDNA3.1-SND1-IT1 + mimics NC or pcDNA3.1-SND1-IT1 + miR-1245b-5p mimics. It was found that, the expression of USP3 was evidently increased by SND1-IT1 overexpression, and was then partially reversed by miR-1245b-5p overexpression (Fig. 4J). The enrichment of USP3 under different conditions was detected by q-PCR in GES-1 cells after RNA pull-down assay. We found that USP3 was highly enriched in Bio-miR-1245b-5p-WT group, and the abundance of USP3 was enhanced after the overexpression of SND1-IT1, indicating the binding between USP3 and miR-1245b-5p is strengthened by SND1-IT1 overexpression (Fig. 4K). In 293 T cells, dual-luciferase reporter assay was performed to detect the luciferase activity under different conditions. The results showed that the luciferase activity was decreased after miR-1245b-5p was overexpressed, which was recovered after co-transfection with pcDNA3.1-SND1-IT1 (Fig. 4L). To sum up, SND1-IT1 competitively adsorbs miR-1245b-5p to up-regulate USP3 expression.

### SND1-IT1 recruits DDX54 to facilitate USP3 mRNA stability

**Fig. 5:**
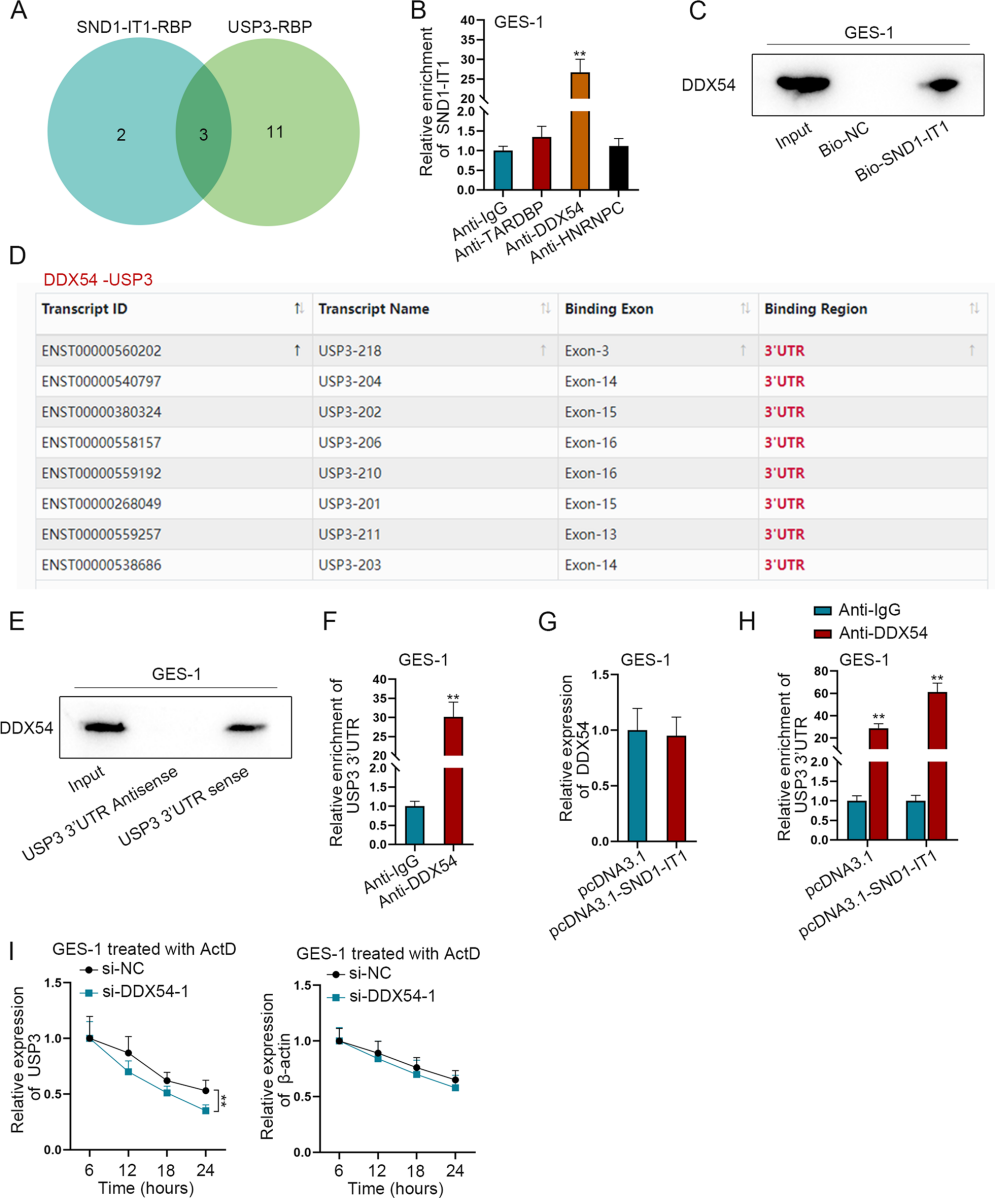
SND1-IT1 recruits DDX54 to facilitate USP3 mRNA stability. **A** The Venn diagram showed the RBPs (TARDBP, DDX54 and HNRNPC) binding to both SND1-IT1 and USP3, which are predicted by starBase. **B** In GES-1 cells, the interaction of SND1-IT1 with RBP candidates was detected by RIP assay. **C** RNA pull-down assay detected the interaction of SND1-IT1 with DDX54. **D** DDX54 was predicted to bind to USP3 3′UTR by using starBase. **E** In GES-1 cells, western blot was used to verify the binding between USP3 3′UTR and DDX54 after RNA pull-down assay. **F** In GES-1 cells, RIP assay was used to verify the binding between USP3 3′UTR and DDX54 after. **G** The expression of DDX54 in GES-1 cells was measured by q-PCR after the overexpression of SND1-IT1. **H** In GES-1 cells, the binding between USP3 3′UTR and DDX54 was detected by RIP assay before and after SND1-IT1 overexpression. **I** USP3 and β-actin half-lives were measured by q-PCR after DDX54 was interfered. ^**^P < 0.01

The results of rescue experiment showed that USP3 expression increased by SND1-IT1 overexpression was partially reversed by overexpressed miR-1245b-5p. Therefore, we speculated SND1-IT1 might regulate USP3 expression through other ways. Literature indicated that lncRNA might regulate downstream mRNA by recruiting RBP [23], thus we speculated that SND1-IT1 might promote USP3 expression via recruiting a RBP. The RBPs (TARDBP, DDX54 and HNRNPC) which can bind to both SND1-IT1 and USP3 were predicted by starBase under the condition of Pan-Cancer ≥ 15, ClusterNum ≥ 30 and ClipExpNum ≥ 5 (Fig. 5A). Afterwards, RIP assay followed by q-PCR was performed in GES-1 cells to detect the enrichments of the candidate RBPs. As evidenced by the higher enrichment, DDX54 had the best binding ability to SND1-IT1 (Fig. 5B). Therefore, DDX54 was chosen for further study. RNA pull-down assay followed by western blot was performed to detect the interaction of SND1-IT1 with DDX54. It was further proved that DDX54 can bind to SND1-IT1 (Fig. 5C). DDX54 was then predicted to bind to USP3 3′UTR by starBase (Fig. 5D). Next, RNA pull-down and RIP assays in GES-1 cells was used to verify the binding condition between USP3 3’UTR and DDX54. The results proved the interaction (Fig. 5E, F). The expression of DDX54 in GES-1 cells was then measured by q-PCR after SND1-IT1 overexpression. The results showed that DDX54 expression remained almost unchanged after overexpression (Fig. 5G). The binding between USP3 3′UTR and DDX54 was detected by RIP assay in GES-1 cells. It was shown that the binding of DDX54 with USP3 3’UTR was enhanced after SND1-IT1 overexpression (Fig. 5H). The interference efficiency of si-DDX54-1/2/3 was detected by q-PCR and western blot (Additional file 3: Fig. S3B, C). Due to its higher efficiency, si-DDX54-1 was selected for follow-up experiments. Next, we performed q-PCR to explore the effect of DDX54 on USP3 mRNA stability in GES cells treated with transcription inhibitor actinomycin D (ActD). USP3 and β-actin half-lives were measured after DDX54 was interfered. It was shown that USP3 half-life was shortened (Fig. 5I). The above experimental outcomes suggested that SND1-IT1 can promote USP3 mRNA stability via DDX54.

### SND-IT1 induces the malignant transformation of GES-1 cells via USP3

**Fig. 6:**
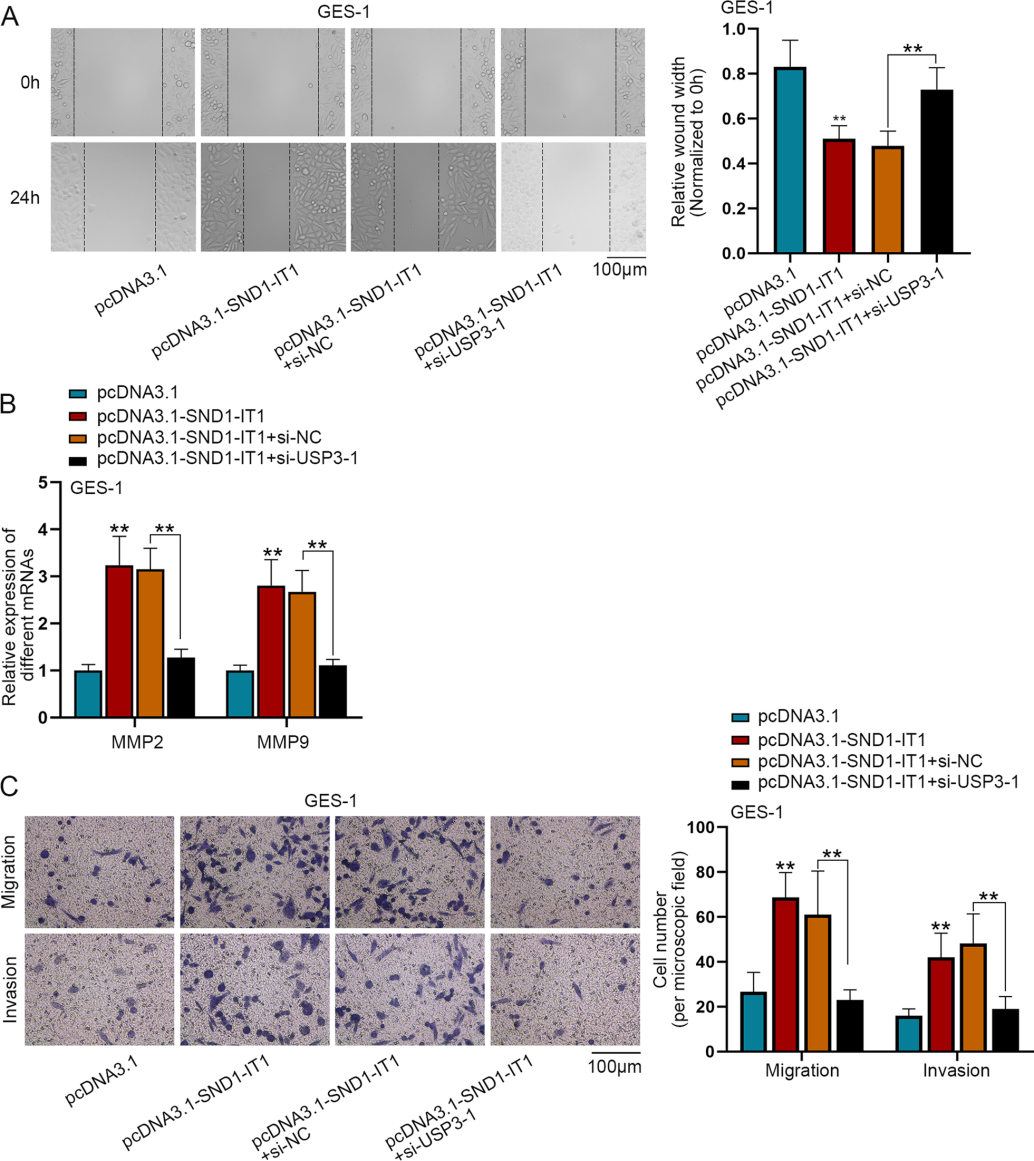
SND-IT1 induces the malignant transformation of GES-1 cells via USP3. GES-1 cells were transfected with pcDNA3.1, pcDNA3.1-SND1-IT1, pcDNA3.1-SND1-IT1 + si-NC or pcDNA3.1-SND1-IT1 + si-USP3-1. **A** Wound healing assay determined the GES-1 cell migration. **B** The expression of MMP2 and MMP9 was confirmed by q-PCR in GES-1 cells. **C** In GES-1 cells, transwell assay was conducted to confirm the cell migration and invasion. ^**^P < 0.01

In this section, we explored whether SND1-IT1 regulates the malignant transformation of GES-1 cells by USP3. Firstly, the interference efficiency of si-USP3-1/2/3 was detected by q-PCR (Additional file 3: Fig. S3D). Due to higher efficiencies, si-USP3-1 and si-USP3-2 were selected for follow-up experiments. Afterwards, experiments were conducted in GES-1 cells to detect cell migration and invasion after transfection with pcDNA3.1, pcDNA3.1-SND1-IT1, pcDNA3.1-SND1-IT1 + si-NC or pcDNA3.1-SND1-IT1 + si-USP3-1 respectively. Wound healing assay was performed to determine the cell migration. It was shown that cell migration was promoted by SND1-IT1 up-regulation, and was then totally reversed by USP3 inhibition (Fig. 6A). The expressions of invasion-related factors, MMP2 and MMP9 were confirmed by q-PCR to detect cell invasion in transfected cells. The results showed that cell invasion promoted by SND1-IT1 overexpression was totally recovered by USP3 depletion (Fig. 6B). Transwell assay was then conducted to assess cell migration and invasion. It was unmasked by the result that, the promoted cell migration and invasion caused by SND1-IT1 overexpression was totally reversed by USP3 interference (Fig. 6C). Taken together, SND-IT1 induces the malignant transformation of GES-1 cells via USP3.

### USP3 mediates SNAIL1 deubiquitination

**Fig. 7:**
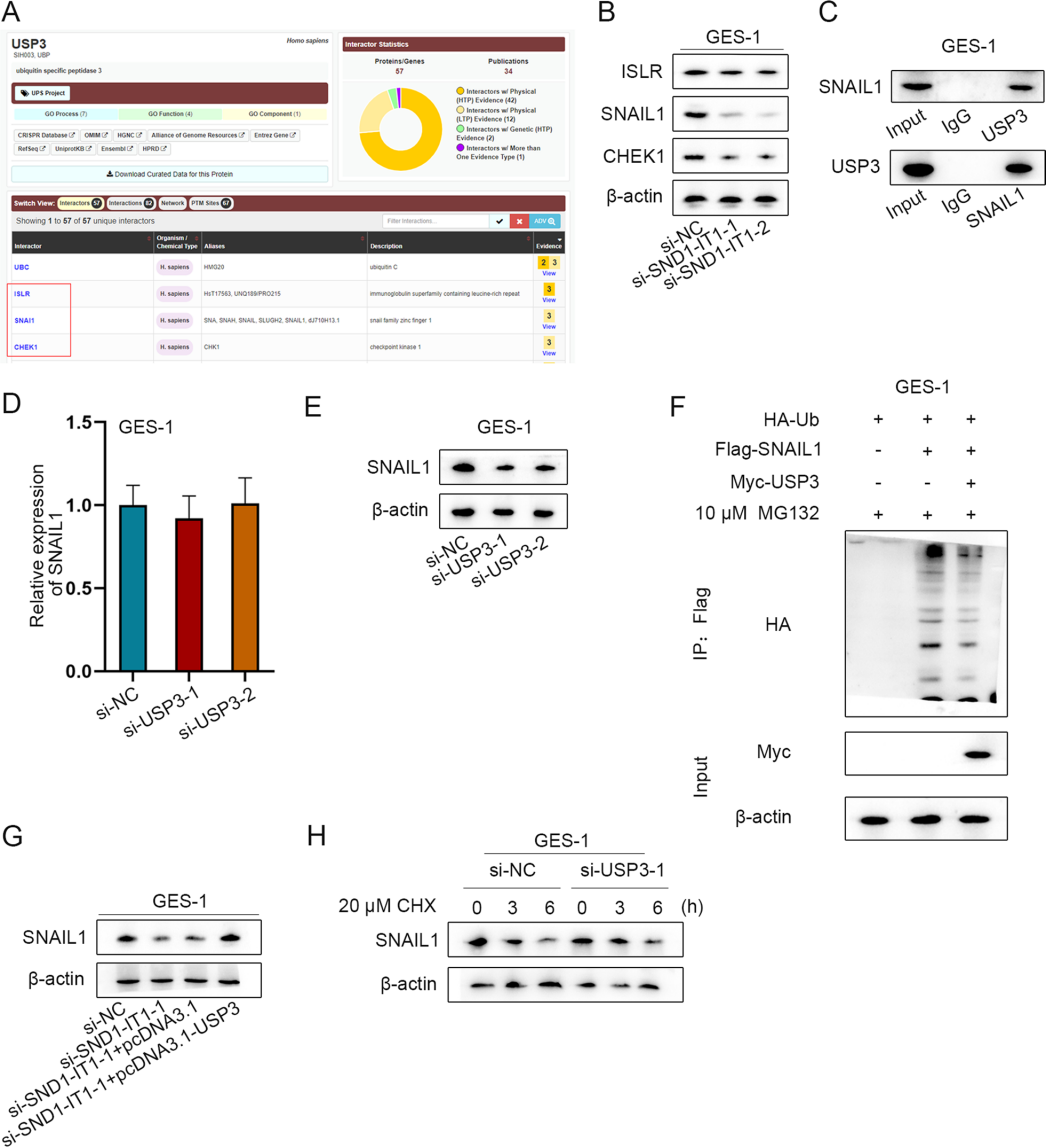
USP3 mediates SNAIL1 deubiquitination. **A** The protein which can interact with USP3 was predicted by BioGrid database (https://thebiogrid.org/) under the condition of Evidence ≥ 3. **B** The protein levels of the candidates was analyzed after SND1-IT1 down-regulation in GES-1 cells. **C** USP3 and SNAIL1 combination was verified by Co-IP assay. **D**, **E** In GES-1 cells, SNAIL1 RNA and protein levels after USP3 knockdown were detected by q-PCR and western blot respectively. **F** SNAIL1 ubiquitination level under different conditions was detected by IP-western blot assay. G. SNAIL1 protein level was detected by western blot assay after the transfection of si-NC, si-SND1-IT1-1, si-SND1-IT1-1 + pcDNA3.1 or si-SND1-IT1-1 + pcDNA3.1-USP3. H. Western blot assay was utilized to measure the SNAIL1 protein level under different time points after addition with CHX

According to literature review, USP3 can mediate protein deubiquitination by acting as a deubiquitinase to promote protein expression and regulate cancer progression [24]. The protein which can interact with USP3 was predicted by BioGrid database under the condition of Evidence ≥ 3. Three proteins were screened out: ISLR, SNAIL1 and CHEK1 (Fig. 7A). According to previous literatures, ISLR promotes colorectal cancer (CRC) progression [25]; SNAIL1 promotes GC cell proliferation and migration [26]; and CHEK1 is up-regulated in lung cancer and inhibits the radiotherapy sensitivity of GC cells [27]. The interference efficiency of si-SND1-IT1-1/2/3 was confirmed by q-PCR in GES-1 cells (Additional file 1: Fig. S3E). Due to higher efficiencies, si-SND1-IT1-1 and si-SND1-IT1-2 were selected for follow-up experiments. The protein level of the candidates was analyzed by western blot after SND1-IT1 down-regulation. As the protein level of SNAIL1 was decreased evidently, SNAIL1 was selected for follow-up assays (Fig. 7B). The combination between USP3 and SNAIL1 was verified by Co-IP assay (Fig. 7C). In GES-1 cells, SNAIL1 mRNA and protein levels were detected by q-PCR and western blot respectively after USP3 ablation. It was shown that the mRNA level of SNAIL1 remained unchanged, while its protein level was significantly decreased (Fig. 7D, E). Subsequently, SNAIL1 ubiquitination level under different conditions was detected by IP-western blot assay in cells treated with MG132, an inhibitor of protein degradation, at 10 µM for 24 h [28]. The outcome showed that USP3 mediated the deubiquitination of SNAIL1 (Fig. 7F). The overexpression efficiency of pcDNA3.1-USP3 was shown in Additional file 3: Fig. S3F. SNAIL1 protein level under different conditions was detected by western blot assay. The results showed that SNAIL1 protein level was decreased when SND1-IT1 was interfered, and was then recovered totally when USP3 was overexpressed (Fig. 7G). Finally, western blot assay was utilized to measure the SNAIL1 protein level under different time points after addition with chlorhexidine (CHX), a protein synthesis inhibitor. CHX treatment concentration was 20 μM [28]. The result displayed that, USP3 ablation inhibited SNAIL1 protein degradation (Fig. 7H). To sum up, USP3 mediates SNAIL1 deubiquitination.

### Exosomal SND1-IT1 is carcinogenic in vivo

**Fig. 8:**
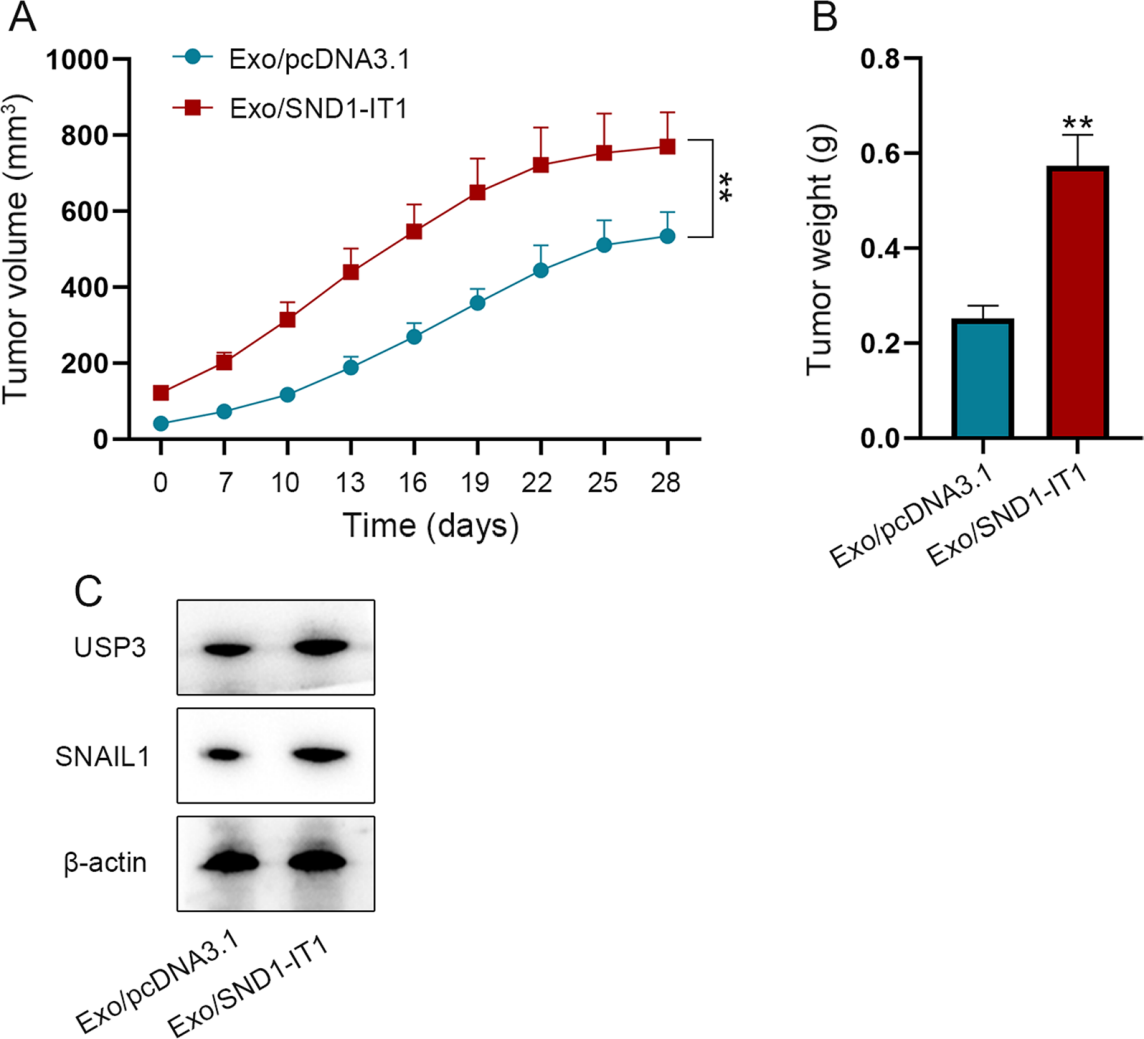
Exosomal SND1-IT1 is carcinogenic in vivo. **A** Tumor volume from 0 to 28 days was measured in nude mice. **B** 28 days after subcutaneous injection, the weight of tumor tissue in nude mice was measured. **C** After 28 days of subcutaneous tumor formation in nude mice, the protein levels of USP3 and SNAIL1 were detected by western blot. ^**^P < 0.01

Outcomes from Figs. 3–7 have indicated that in in vitro experiments, exosomal SND1-IT1 induces GES-1 malignant transformation. Thus, we next verified the carcinogenic role of exosomal SND1-IT1 in vivo. Nude mice received subcutaneous injection of exosomes Exo/pcDNA3.1 or Exo/SND1-IT1. Seven days after the injection, tumor volume was measured every 3 days thereafter [29]. Volume of xenografts from 0 to 28 days of subcutaneous tumorigenesis in nude mice was measured. Compared to the control group, the tumor volume was higher in nude mice injected with Exo/SND1-IT1 (Fig. 8A). Twenty-eight days after injection, the weight of xenografts resected from nude mice was measured. Likewise, the tumor weight was higher in the Exo/SND1-IT1 group than that in Exo/pcDNA3.1 group (Fig. 8B). Western blot assay was conducted to detect the protein levels of USP3 and SNAIL1 in xenografts. The results showed that the protein levels in Exo/SND1-IT1 group were increased obviously compared to the control group (Fig. 8C).

## Discussion

GC, as a prevalent and heterogeneous disease, features unsatisfying prognosis [30, 31]. Despite great progress made in GC treatment, the proper therapeutic strategies for GC still remain to be explored. Biological and molecular markers are of great value in the diagnosis, prognosis and treatment of malignant tumors. For instance, metadherin has the potential to function as a diagnostic and prognostic marker in CRC [32]; neuropilin-1 and angiopoietin-2 serve as markers for hepatocellular carcinoma [33]; miR-150 expression can be used to predict imatinib response chronic myeloid leukemia patients [34]; and the expression of Oct4 is correlated with GC progression [35]. In order to improve the treatment of GC, we conducted the study on the specific mechanism of lncRNA SND1-IT1 and its potential as a biomarker in GC cells.

LncRNAs have been reported to participate in the regulation of disease development and play a critical role in various biological functions [36]. As indicated in the previous studies, lncRNA SND1-IT1 has been reported to modulate various cancers. For instance, SND1-IT1 accelerates the proliferation and migration of osteosarcoma via sponging miR-665 to up-regulate POU2F1 expression [18]. In addition, SND1-IT1 is involved in rat myocardial ischemia/reperfusion injury via regulating miR-183-5p [37].

**Fig. 9:**
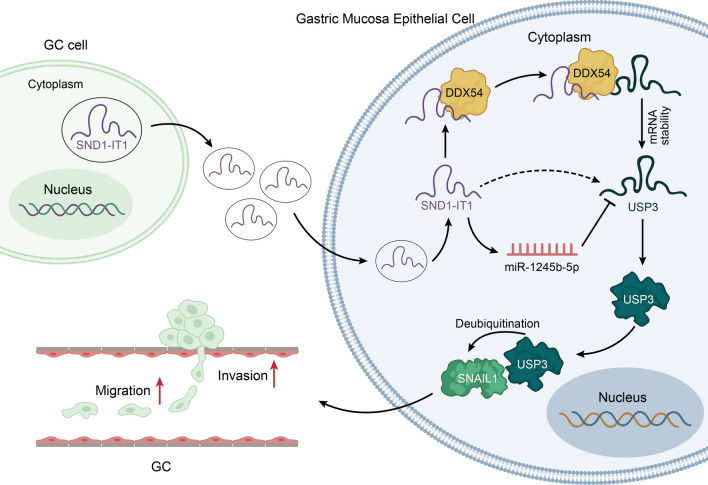
Exosome-mediated SND1-IT1 from GC cells interacts with miR-1245b-5p and DDX54 to up-regulate the expression of USP3, which mediates SNAIL1 deubiquitination to enhance the malignant transformation of gastric mucosa epithelial cell

It has been reported that in TME, cancer cells can induce normal cells to promote carcinogenesis [15]. In our research, we co-cultured GC cells and gastric mucosa epithelial cell GES-1 at first. It was found that the malignant transformation of GES-1 cells was promoted by exosomes secreted from GC cells. With the help of GEO database (GSE153413), lncRNAs which were highly expressed in the exosomes from the plasma of GC patients were analyzed. It was found that lncRNA SND1-IT1 was obviously overexpressed in exosomes. Therefore, SND1-IT1 was chosen as the focus of the present study. Next, we conducted experiments and discovered that exosomal SND1-IT1 can induce the malignant transformation of GES-1 cells. In order to figure out the specific mechanism of SND1-IT1 underlying its promoting effect on canceration, we conducted experiments for exploration after bioinformatics prediction. It was verified that SND1-IT1 can promote USP3 mRNA expression through competitively adsorbing miR-1245b-5p. Meanwhile, SND1-IT1 was proved to recruit DDX54 to improve USP3 mRNA stability. As USP3 can mediate protein deubiquitination, we used BioGrid to predict the proteins which can interact with USP3. Further assays were performed to confirm that USP3 modified deubiquitination of SNAIL1 and facilitated its protein expression. In the end, animal experiments were conducted to verify that exosome-mediated SND1-IT1 can promote cancer progression in vivo. The graphical abstract illustrated the underlying mechanism. As shown in Fig. 9, exosomal lncRNA SND1-IT1 secreted from GC cells could competitively absorb miR-1245b-5p and simultaneously recruit DDX54 to up-regulate USP3 expression, thus mediating SNAIL1 deubiquitination and inducing the malignant transformation of GES-1 cells.

## Conclusion

Our study firstly manifested the promoting effect of exosomal SND1-IT1 on gastric mucosa cell malignant transformation. It has firstly been discovered in the present study that SND1-IT1 can competitively adsorb miR-1245b-5p and recruit DDX54 to facilitate USP3 expression. Moreover, our study suggested that USP3 can mediate the deubiquitination of SNAIL1 for the first time. However, our report can be improved in many aspects. Because of the lack of fund support and human power, we could not conduct clinical trials. In addition, only one type of GC cell line was utilized in experiments, which limits the stringency of our report. In the future, we will probe into the clinicopathological relevance and validate the results in other GC cell lines in the further study.

## Supplementary Information

**Additional file 1: Figure S1.** Differential expression analysis of candidate lncRNAs in GC. A-I. UALCAN database (http://ualcan.path.uab.edu/index.html) was utilized to analyze the expression of lncRNAs in STAD.

**Additional file 2: Figure S2.** A-B. The overexpression efficiency of pcDNA3.1-SND1-IT1 was determined in AGS and GES-1 cells by q-PCR. C-J. The expressions of mRNAs in STAD were analyzed by starBase database

**Additional file 3: Figure S3.** A. In GES-1 cells, the overexpression efficiency of miR-1245b-5p mimics was detected by q-PCR. B-C. In GES-1 cells, q-PCR and western blot assays were conducted to measure the interference efficiency of si-DDX54-1/2/3. D. The interference efficiency of si-USP3-1/2/3 was confirmed by q-PCR in GES-1 cells. E. The interference efficiency of si-SND1-IT1-1/2/3 was confirmed by q-PCR in GES-1 cells. F. The overexpression efficiency of pcDNA3.1-USP3 was measured by q-PCR in GES-1 cells. ^**^P < 0.01

## Data Availability

Not applicable.

## Abbreviations

GC: Gastric cancer
lncRNAs: Long non‑coding RNAs
miRNAs: MicroRNAs
mRNAs: Messenger RNAs
RBPs: RNA-binding proteins
NC: Negative control
cDNA: Complementary DNA
IgG: Immunoglobulin G
IP: Immunoprecipitation
SD: Standard deviation
ANOVA: Analysis of variance
