# Supplementary figures and images for: Exosome-mediated lncRNA SND1-IT1 from gastric cancer cells enhances malignant transformation of gastric mucosa cells via up-regulating SNAIL1
Source: J Transl Med. 2022 Jun 23;20:284. doi: 10.1186/s12967-022-03306-w (PMC9229915; doi:10.1186/s12967-022-03306-w)

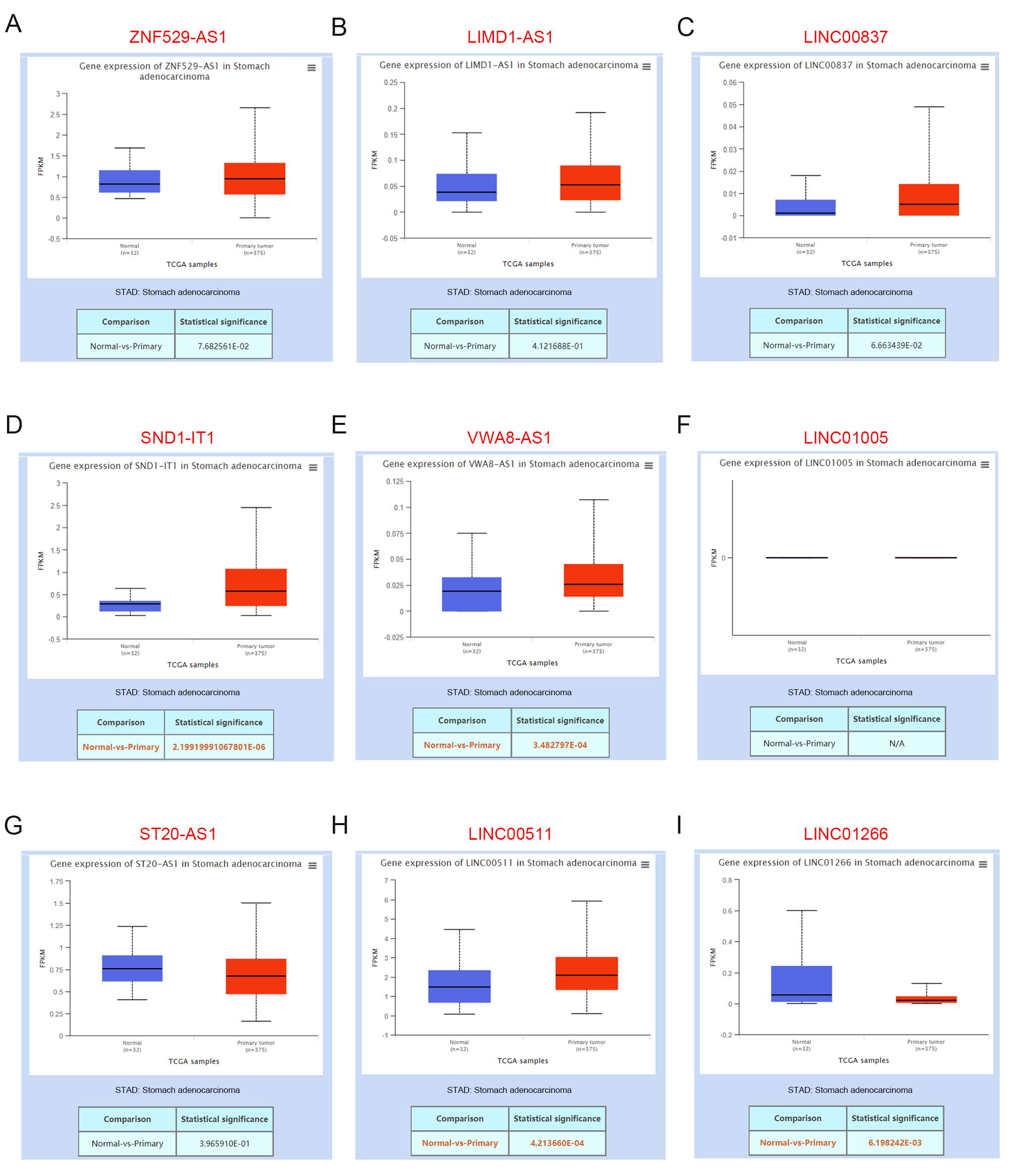

Supplement: Supplementary file 1 — Additional file 1: Figure S1. Differential expression analysis of candidate lncRNAs in GC. A-I. UALCAN database (http://ualcan.path.uab.edu/index.html) was utilized to analyze the expression of lncRNAs in STAD. [file 12967_2022_3306_MOESM1_ESM.tif]

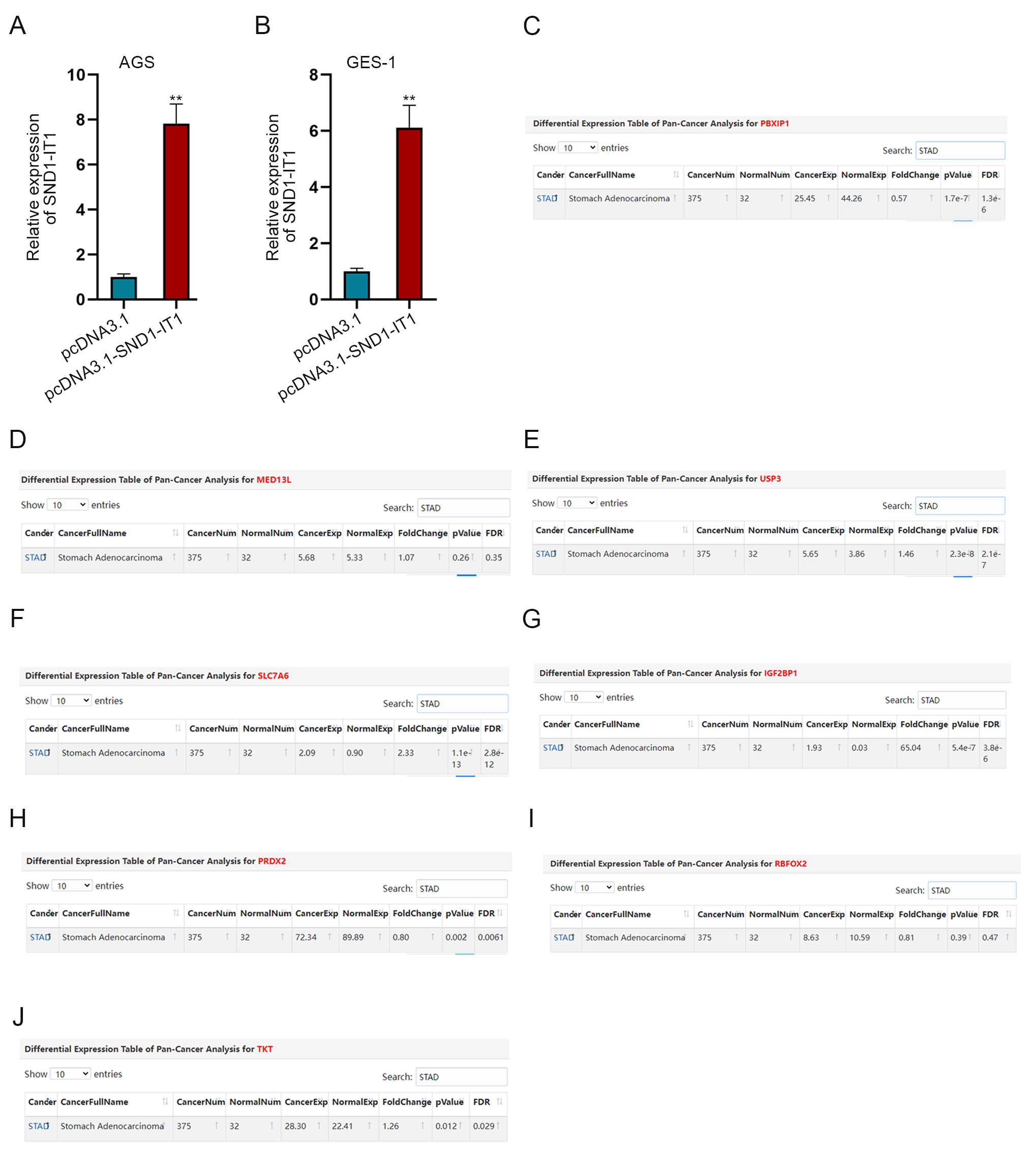

Supplement: Supplementary file 2 — Additional file 2: Figure S2. A-B. The overexpression efficiency of pcDNA3.1-SND1-IT1 was determined in AGS and GES-1 cells by q-PCR. C-J. The expressions of mRNAs in STAD were analyzed by starBase database [file 12967_2022_3306_MOESM2_ESM.tif]

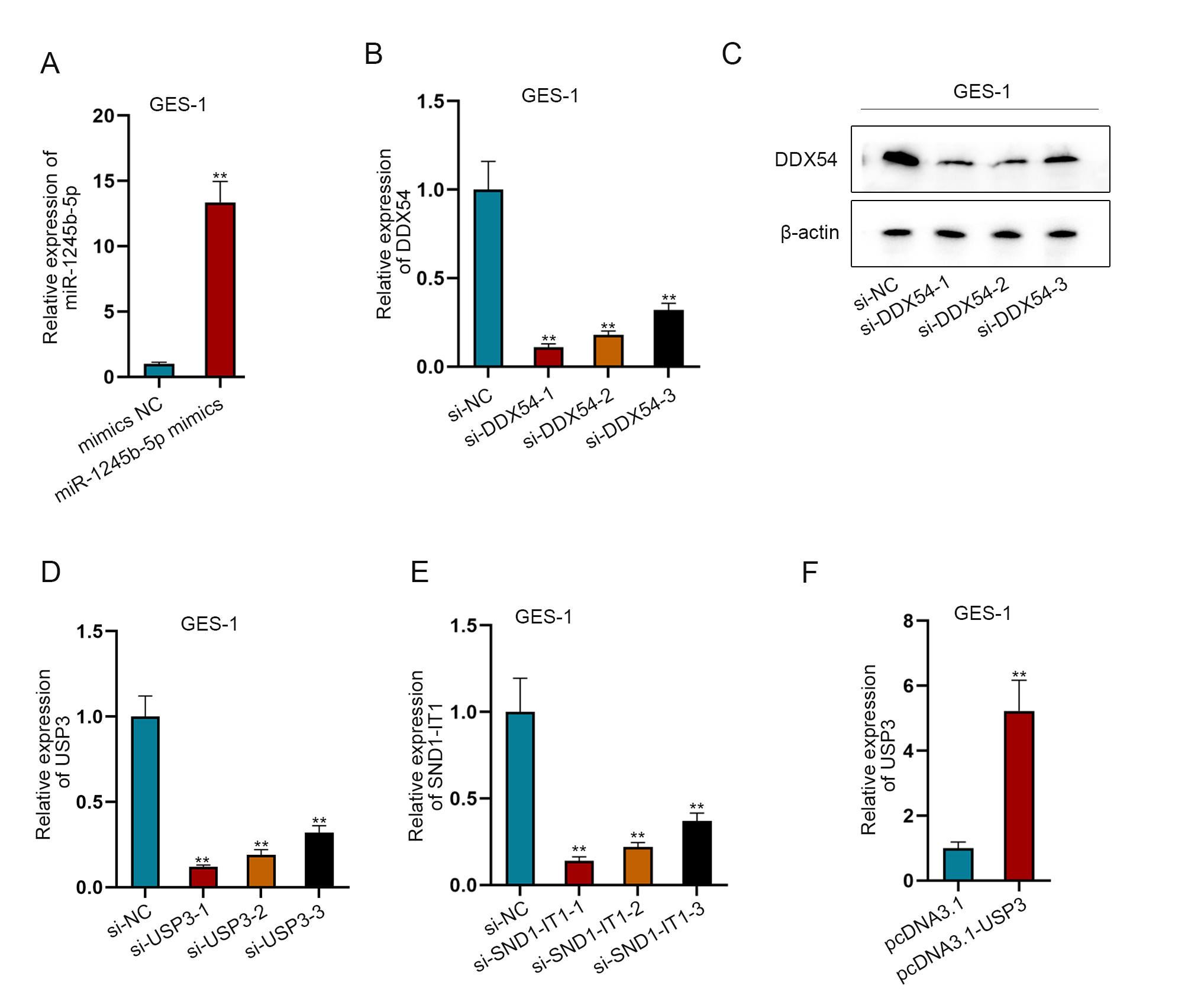

Supplement: Supplementary file 3 — Additional file 3: Figure S3. A. In GES-1 cells, the overexpression efficiency of miR-1245b-5p mimics was detected by q-PCR. B-C. In GES-1 cells, q-PCR and western blot assays were conducted to measure the interference efficiency of si-DDX54-1/2/3. D. The interference efficiency of si-USP3-1/2/3 was confirmed by q-PCR in GES-1 cells. E. The interference efficiency of si-SND1-IT1-1/2/3 was confirmed by q-PCR in GES-1 cells. F. The overexpression efficiency of pcDNA3.1-USP3 was measured by q-PCR in GES-1 cells. **P < 0.01 [file 12967_2022_3306_MOESM3_ESM.tif]
